# Chronic cough due to occupational factors

**DOI:** 10.1186/1745-6673-1-3

**Published:** 2006-02-02

**Authors:** David A Groneberg, Dennis Nowak, Anke Wussow, Axel Fischer

**Affiliations:** 1 Division of Allergy Research, Otto-Heubner-Centre, Charité School of Medicine, Free University and Humboldt-University, 13353 Berlin, Germany; 2 Institute and Outpatient Clinics for Occupational and Environmental Medicine, University of Munich, 80336 Munich, Germany; 3 Institute of Occupational Medicine, University of Lübeck, 23538 Lübeck, Germany

## Abstract

Within the large variety of subtypes of chronic cough, either defined by their clinical or pathogenetic causes, occupational chronic cough may be regarded as one of the most preventable forms of the disease. Next to obstructive airway diseases such as asthma or chronic obstructive pulmonary disease, which are sometimes concomitant with chronic cough, this chronic airway disease gains importance in the field of occupational medicine since classic fiber-related occupational airway diseases will decrease in the future.

Apart from acute accidents and incidental exposures which may lead to an acute form of cough, there are numerous sources for the development of chronic cough within the workplace. Over the last years, a large number of studies has focused on occupational causes of respiratory diseases and it has emerged that chronic cough is one of the most prevalent work-related airway diseases. Best-known examples of occupations related to the development of cough are coal miners, hard-rock miners, tunnel workers, or concrete manufacturing workers.

As chronic cough is often based on a variety of non-occupational factors such as tobacco smoke, a distinct separation into either occupational or personally -evoked can be difficult. However, revealing the occupational contribution to chronic cough and to the symptom cough in general, which is the commonest cause for the consultation of a physician, can significantly lead to a reduction of the socioeconomic burden of the disease.

## Introduction

Not only since its association to exposed firefighters after the collapse of the World Trade Center [1] or work-related stress [2] is chronic cough discussed as a potential work-related disease. Chronic cough is a common disease [3] and the entity occupation-related chronic cough could be defined as a disease state that is characterized bythe presence of cough for a period longer than three months per year which is caused by an occupational exposure to an airway irritant. A cessation of the symptoms should be observed when the occupational exposure is discontinued.

Pathophysiologically, the disease is most probably dependant on the activation of two different types of sensory receptors in the respiratory innervation: C-fibre receptors with non-myelinated afferent nerve fibres, and rapidly adapting receptors (RARs) which are localized to thin myelinated A. fibres [4,5]. Also, an increased expression of transient receptor potential vanilloid-1 has been recently demonstrated in airway nerves of patients with chronic cough [6,7] and airway nerves and their mediators in general are likely to play an important role in the pathology of cough and airway inflammation [8-11].

It has recently been shown that COPD and to a lesser extent also asthma and rhinitis are associated with a substantially shortened work life [12]. Workplace-related chronic cough may often be found concomitant with obstructive respiratory diseases such as occupational asthma or chronic obstructive bronchitis (COPD) and in general with work-related airflow limitation which occurs due to an exposure to allergens, toxins or organic dusts such asflax, sisal, cotton (byssinosis), or hemp.

In contrast to the current knowledge on the pathophysiology and epidemiology of occupational asthma, rhinitis or COPD, which were assessed by numerous experimental [13-16] and clinical [17-21] studies, workplace-related chronic cough has never been directly focused by epidemiological or experimental studies. However, due to the simple definition and the high prevalence, cough symptoms were included to many questionnaires on occupational asthma and COPD and therefore, reviewing the literature also reveals a major role of cough as a work-related disease.

### Evidence for an occupational contribution to Chronic Cough

#### Animal studies

In contrast to the large number of studies and animal models on asthma and COPD [22-26], there are only a few reports on animal models of cough. Therefore, a clearly established experimental link only exists for occupational causes of asthma and COPD, including allergens, irritants such as TDI [27] or other noxious gases such as NO2 [28], SO2 [29] or ozone [30,31], Due to the need for a suitable model which offers the closest proximity to the human condition [32], ongoing research still aims to establish a model which can then be examined for the influence of occupational triggers of cough. Existing animal models of cough include guinea pigs [33,34], rats and mice [35] but within each of these species, significant limitations concerning the neurophysiology of the cough reflex have been found [32]. In contrast to epidemiological studies for the relation between workplace exposure and respiratory symptoms in workers, which always include cough as symptom, the large number of studies on the respiratory effects of occupational noxious substances such as silica or vanadium in animals [36,37] did not include a cough assessment due to methodological difficulties.

#### Epidemiologic studies

It is generally accepted that active of passive exposure to tobacco smoke can be a factor leading not only to COPD but also to cough. Due to the still large prevalence of active and passive exposure to smoke, it is difficult to isolate the effects of occupational exposures on the development and progression of cough.

However, there is increasing evidence present that demonstrates the involvement of specific occupational substances in the development of chronic cough. In this respect, a number of studies have addressed the associations between the disease and different occupational exposures:

#### Exposure in mines

A variety of studies has examined the relation between occupational exposure in mines and chronic cough. In a study including 970 coal-miners without pneumoconiosis, chronic cough was found in 50.5% of a group of. In 14.7% of these individuals the cough was not connected to chronic phlegm. It was therefore concluded that this subtype of non productive chronic cough displays a common disease within coal miners and that it may not be neglected in the natural history of chronic bronchitis in coal-miners. The changes in lung function were reported to be similar in non-productive and productive chronic cough [38].

The relation between chronic cough, coal mining and coal miners' pneumoconiosis (CMP) was examined in 205 miners with simple CWP and 289 without CWP, as a coincidence of pneumoconiosis and chronic cough was suggested before but had never been proofed. Logistic regression and multiple linear regression analysis were used to assess the independent effect of CWP and the miners with CWP had higher prevalences of respiratory symptoms. Also, after the adjustment for smoking, age, and years of underground mining work, CWP was associated with an increased risk and it was thus concluded that the simple coal miners' pneumoconiosis is a contributor to a significantly increased risk to develop chronic cough [39].

A further study on 242 Utah underground coal miners (mean age: 56 years) who had spent a mean of 29 years in the coal-mining industry revealed that exposure to coal mine dust had a significant influence in causing the symptom complex of chronic cough and sputum production, and coal worker's pneumoconiosis. There was a significant association among nonsmoking subjects between an increasing exposure to the coal dust and coal worker's pneumoconiosis, but not for changes in the pulmonary function [40]. A later prospective study assessed whether the presence of chronic cough and chronic phlegm predicts 5-year cigarette smoking cessation in cohort of 1.118 US male coal miners. It was shown that the presence of chronic cough and phlegm initially was inversely associated with cigarette smoking cessation. However, lung function (FVC and FEV1) impairment was positively associated with cigarette smoking cessation but did not reach statistical significance [41]. In another study, the combined effects of exposure to gold mining dust with a high content of free silica and the role of tobacco smoking were examined among 2209 South African gold miners and 483 nonminers and it was shown that tobacco smoke strongly potentates the effect of the dusts [42].

A recent study provided further detailed information on the relation between chronic cough and coal mining and discriminated between exposure to silica, asbestos, and coal dusts [43]. In total, 220 workers exposed to silica, 277 workers exposed to asbestos, and 511 workers exposed to coal from three different industries in China were examined and significantly higher prevalence of chronic cough was found in workers with pneumoconiosis than those without, irrespective of dust type. Workers with stages II and III silicosis had increased rates for chronic cough and worse pulmonary function when compared to workers with equivalent coal workers' pneumoconiosis or asbestosis [43].

Chronic cough and phlegm and their relation to surface and underground mining were also examined in a cross sectional survey of 1363 Australian men [44]. Overall, the prevalence was 14%. Smoking and age exerted strong effects on the development of symptoms and after control of confounding by these two factors, it was estimated that compared with a lifetime non-miner, the odds ratio (OR) of chronic cough and phlegm was 1.8 (95% confidence interval, 1.0–3.3) for one to nine years underground mining gold, 2.5 (1.2–5.2) for 10–19 years, and 5.1 (2.4–10.9) for more than 20 years. Whereas surface mining had only small empirical effects (OR = 1.3; 95% CI, 0.6–2.5), underground mining of other minerals than gold was also associated with chronic cough and phlegm (OR = 5.1; 95% CI, 1.1–25.0) In summary it was estimated that the proportion of cases of chronic cough and phlegm in working underground miners due to occupational factors is about 50% [44].

### Cement

Dust arising from cement production contains various substances and before its abolishment, asbestos was regarded as the major factor leading to occupational diseases in the cement industry [45-47]. However, dusts contain crystalline silica which is also generated in granite, glass, and mining industries and which may lead to chronic cough [48,49] and significant associations between exposure to concrete dust and decreased lung function (FEV(1)/FVC ratio, MMEF) have been reported in concrete workers, independent of smoking habits and of a history of allergy [50] and chronic cough was also among the major health problems in other cement worker studies [51]. For instance, a cross-sectional survey of 348 Portland cement workers in Jordan [52] revealed that chronic cough was the most prevalent respiratory problem (prevalence of 18.7%) while overall pulmonary function indices were reported to be normal. As with other studies, the rate of cough increased proportionally to the number of cigarettes smoked per day. In this study, it was concluded that the inhalation of cement dust irritates the respiratory tree and causes chronic cough without markedly affecting lung function, while smoking aggravates this effect [52].

As the seriousness of pulmonary effects has not been consistently associated with the degree of exposure to cement dust and the regular use of appropriate personal protective equipment, is still not always adequately used, a study explored the prevalence of chronic cough and other respiratory symptoms such as chronic phlegm among Portland cement workers. In comparison to non-exposed individuals, a higher percentage of the cement-exposed workers reported chronic cough (30% vs. 10 %). Also, respiratory functions such as VC, FVC, FEV(1), FEV(1)/FVC, FEV(1)/VC were significantly lower in the exposed workers if compared to the unexposed workers. These differences were not explained by age, or smoked pack-years and it was concluded that adverse respiratory health effects such as chronic cough were most likely caused by the exposure to cement dust [53]. These findings are supported by further studies on the relationship between occupational Portland cement dust exposure and respiratory health which reported a higher prevalence of chronic respiratory symptoms such as chronic cough in exposed than in control workers [54].

Exposure to cement dusts is even a larger problem in non-industrialized countries as personal protection equipment is limited [55]. A study in Nigerian cement workers revealed an increased prevalence of chronic cough due to cement dust exposure (dust level in the cement depot was 30.81 mg/m3) in 52 randomly selected, directly exposed cement workers and 24 maintenance workers if compared to a control group. The exposure also lead to a significantly decreased (P less than 0.001) lung function. Furthermore, the quantity of exposure was examined and the more directly exposed cement packers had significantly (P less than 0.05) lower lung function values than less exposed cement loaders [55].

A study in three different sections of cement, yarn, and cigarette factories among 211 nonsmoking male and female workers (21–57 years) vs. 211 healthy nonsmoking and non-exposed male and female subjects (20–57 years) revealed that 24.7% of the dust-exposed subjects developed chronic cough (vs. control 9.0%). The effects of exposure to cement dusts and cotton on the respiratory health of the exposed subjects were relatively more significant (p < 0.001) than that of exposure to tobacco dust (p < 0.05) [56].

To summon up this data on chronic cough and mining, a recent study was conducted in the United States [57]. The long-term health outcomes associated with rapid declines in FEV_1 _observedamong some underground coal miners was determined by evaluating respiratory symptoms including chronic cough and mortality among a group of miners who had earlierdemonstrated accelerated losses of FEV_1 _in the U.S. National Studyof Coal Workers' Pneumoconiosis (NSCWP). The comparison with amatched referent group of miners with relatively stable lung function revealed that there was a greater incidence of chronic cough in the miners' group, indicating an increased risk for dust-exposed workers with rapid declines in FEV_1 _to develop chronic cough and other respiratory symptoms [57].

### Construction workers

Underground and surface construction work has also been reported to be associated with the development of chronic cough. For example, underground construction work is associated to various exposures. Next to oil mist, major exposures include dust, alpha-quartz, and nitrogen dioxide [58,59]. To examine whether underground construction workers exposed to tunneling pollutants have an increased risk of chronic cough and other respiratory symptoms, a group of workers were examined over a follow up period of 8 years and compared to other heavy construction workers. Exposure measurements were carried out to estimate personal cumulative exposure to total dust, respirable dust, alpha-quartz, oil mist, and nitrogen dioxide and it was shown that compared with the reference group the odds ratio for the occurrence of new respiratory symptoms during the follow up period was increased in the tunnel workers [60]. Compared with the reference group, and after adjustment for age and current smoking, the odds ratios for new chronic cough (morning cough or cough during the day) were increased in the tunnel workers. Respiratory symptomswere associated with cumulative exposure to respirable dust, but not with alpha-quartz.

Oil mist has been the focus of two other studies which also demonstrated that this exposure is associated to an increase in chronic cough: In a study at a large French car-making plant, subjects exposed to straight cutting oils had a significantly higher prevalence of chronic cough and/or phlegm than the control (25.7% vs. 16.3%, p = 0.048). The prevalence of cough and/or phlegm was also significantly increased with increasing duration of exposure to straight cutting oils after adjustment on smoking categories [61]. These data were confirmed in a later study, which demonstrated that the prevalence of chronic cough or phlegm was increased greater among oil mist-exposed workers than among controls (odds ratio (OR) 4.64, P = 0.002) [62].

To define the relation between commonly used brick stones and chronic cough, 233 male workers employed in two brick-manufacturing plants (mean duration of employment: 16 years) were examined and compared to 149 matched control workers. There was a significantly higher prevalence of chronic cough () in exposed workers, compared with control workers (31.8% vs. 20.1%, P < 0.05) and the increased frequency was also documented among nonsmokers studied by age and by length of employment, suggesting a work-related effect [63].

In order to relate sewage work to the development of chronic cough and other respiratory diseases, a group of 74 sewage workers were studied by their work stations (closed channels, drainage, other places) and the prevalence of chronic cough was increased in closed channel and drainage workers if compared to controls (range: 41.9–46.2% vs. 14.3%) [64].

### Farming and food industry

The farming and food industry displays a further field of economy in which an increased prevalence of chronic cough may be anticipated, as there are numerous irritant substances arising from the farming and food processing [65].

A series of reports has focused on the association of chronic cough and farming. In this respect, a study was conducted among dairy farmers (n = 265) and a control group of non-exposed subjects (n = 149) to determine whether dairy farming is associated with respiratory symptoms [66]. It was shown that while the prevalence for asthma did not differ significantly, there was strong association to chronic cough, phlegm, and chronic bronchitis. It was also suggested that the combined effect of farming and smoking was synergistic on chronic cough [66]. A larger study which did not discriminate between different farming forms confirmed these findings and reported a prevalence of 24.8 % vs. 11.4 % control) for chronic cough in farm workers [67].

To study the prevalence of chronic cough and other chronic and acute respiratory symptoms in livestock farm workers raising cattle and horses, a cross-sectional study and lung function tests were performed in 236 (169 male and 67 female) subjects [68]. The study demonstrated that there was a significantly higher prevalence of almost all chronic respiratory symptoms in farmers, and the highest prevalence was found for chronic cough in men (27.2%). As with many other studies, a logistic regression analysis indicated significant effects of smoking (P < 0.001). In this study, a large number of male and female livestock farm workers also complained of acute dry cough (52.2%) at the place of work [68].

In contrast to cattle farmers, swine confinement farms are differently exposed. To define in detail the nature and disease determinants in this exposed group, a 5 year prospective study was conducted [69], and it was found that nearly 20% of swine confinement workers reported chronic cough, which was significantly more prevalent compared to a blue collar comparison group. As in livestock farms, acute work place-related cough was much more frequent than chronic cough (87% of confinement workers reported work place-related cough) [69]. Similar findings for chronic cough were also reported by a smaller study [70] and it was also shown for veterinarians, that working more than 20 hr per week in swine confinement buildings increased the odds of occurrence of chronic cough approximately three times [71].

Poultry farming has also been associated with the development of airway diseases and therefore, a group of 343 poultry farmers was studied for the prevalence of acute and chronic respiratory symptoms and lung function changes. Amongst others, there was significantly higher prevalence of chronic cough in the poultry farmers and workers exposed for more than 10 years had significantly higher symptoms prevalences than farmers with shorter exposures (except among female smokers) [72]. An additional study on a group of 57 female workers employed in the processing of poultry food and 51 non-exposed control workers showed similar results with the highest prevalence for chronic cough (49.1%), followed by dyspnea (43.9%), rhinitis (38.6%) [73].

A further report examined the respiratory health of Californian rice farmers who are occupationally exposed to various agents such as inorganic dusts and smoke from burning of agricultural waste [74]. Here, chronic cough was reported by 7.1% and was associated with reported hours per year burning rice stubble, while the prevalence for other diseases were 6.3% for chronic bronchitis and 7.1% for physician-diagnosed asthma, indicating prevalences among rice farmers [74]. A high prevalence for chronic cough was also reported for cash grain farmers in Ohio with an overall design-adjusted prevalence corresponding 95% confidence intervals (CIs) of 9.4% (CI: 7.6–11.1%) [75].

As hemp workers with a long exposure to dust were reported to develop chronic respiratory symptoms including cough, a 7 years of follow-up study was performed. Among the hemp workers, a significantly higher occurrence of chronic cough was found which was concomitant with a significantly larger annual decline of forced expiratory volume in 1 second (FEV1.0) [76]. Similarly, a study was conducted in workers exposed to flour dust in a flour mill. Here, 26.0% of the exposed subjects presented with chronic cough, indicating a strong association between exposure to flour dusts and the prevalence of chronic cough [77]. Flour dusts are also associated with a large number of occupational diseases among bakers. Parallel to the findings in flour mill workers, it was shown that in bakers a greater prevalence of chronic cough and sputum can be found [78].

The influence of grain processing on the prevalence of respiratory diseases was also studied in dock workers who load grain cargoes and dock workers who work in grain elevators. It was found that the prevalences of chronic cough were similar in both group and the risks to develop symptoms were significantly increased for the dock workers if compared to non-exposed workers from the same regions [79].

Next to flour mill workers and grain workers, workers in other food-producing facilities have also been reported to have increased prevalences of chronic cough. In this respect chronic cough was found in potato-processing workers [80] and in spice factory workers [81]. But also, workers who are not as highly exposed to dusts have been reported to develop chronic cough. For instance, vegetable pickers [82] or greenhouse workers [83] were reported to have higher prevalences of chronic cough and also workers who process mushrooms [84-86].

### Wood industry

Whereas exposure to wood dusts is known for occupational diseases such as cancer [87-89], chronic cough has also long been discussed as wood-dust related [90]. For instance, a study on respiratory symptoms in differentially exposed pine sawmill workers revealed adjusted odds ratios for cough at 2.7 (1.2–6.5) for the low, 5.2 (2.1–13.0) for the high 'green dust' and 3.3 (1.4–7.9) for the high 'dry dust' exposure groups. It was concluded that working in pine sawmills is associated with an increased prevalence of cough, next to eye and nose irritation and asthma [91]. Wood dust exposure was also the focus of a study among furniture workers and it was shown that dry cough was the most common illness [92]. Similarly, in a paper mill cohort consisting of workers with high cumulative exposure (n = 313), with medium cumulative exposure (n = 321), and with low cumulative exposure (n = 338) to paper dust, elevated prevalence ratios for chronic cough and chronic phlegm were found after adjustment for smoking and gender [93]. Also, paper recycling is associated with an increased risk for chronic cough, which had the highest prevalence (36.6%) of all respiratory symptoms [94].

### Chemicals

Next to associations between chronic cough and farming or wood dusts, cough may also arise from chronic irritation of respiratory nerves by chemicals and various studies have addressed this question.

For exposure to methylmethacrylate (MMA) it was shown that 20% of subject in the MMA-exposed group had chronic cough compared with 1% in the control [95]. Also, a study among workers of a polyvinylchloride (PVC) factory suggested chronic cough and chronic phlegm may be developed due to exposure to vinyl chloride monomer, PVC dust, PVC thermal degradation products and other non-identified substances [96]. A smaller study among workers who were exposed to aliphatic polyamines in a chemical factory gave further evidence to a relation between exposure to chemicals and chronic cough [97] and a need for stringent measures to protect the respiratory health of industrial workers exposed to aliphatic amines was proposed [97].

Dental technicians are exposed to a large variety of chemicals and as with furriers, these occupations were reported to be associated to chronic cough [98,99].

Next to these exposure in the chemical industry, a variety of other substances including latex [100] was reported to be related to chronic cough and also the surrounding of the work place, i.e., cold houses, may contribute to the prevalence of cough [101].

### General studies

Next to the large amount of studies which focused on a specific occupational background, there are several studies which were conducted in random samples and examined for relationships between occupational exposure chronic respiratory symptoms.

Data analysis from a random sample of 8,515 adults from 6 cities in the eastern and Midwestern United States [102] revealed that 31% of the population was occupationally exposed to dust and 30% of the population was exposed to gas or fumes. After adjusting for smoking habits, gender, age, and city of residence, subjects with either occupational exposure had significantly elevated prevalences of chronic cough. In summary, the data demonstrated that chronic respiratory symptoms and disease can be independently associated with occupational exposures [102].

A longitudinal analysis of the relationship between incidence rates of chronic respiratory symptoms over 13 years and occupational exposure during the first five years of follow-up in 1132 males and 1598 females (19 to 60 years of age) in Cracow, Poland [103] came to similar results [103] and confirmed the results of cross-sectional studies which have indicated deleterious effects of even moderate occupational exposures on respiratory symptoms [103].

Other studies in France [104] Spain [105] confirmed these data. In an un-polluted rural area of North Italy, the effects of occupational exposure in a general population sample (age range of 18 to 64 yr) with 417 participants who reported any exposure to dusts, chemicals, or gases and 1,218 who reported no exposure were examined. In summary, multiple logistic models in the overall group, accounting for age, smoking, and pack-years, demonstrated that self-reported occupational exposure was associated significantly with higher risks for cough (odds ratio: 1.69), suggesting a causal association between occupational exposure and chronic pulmonary diseases such as cough [106].

A recent statement of the American Thoracic Society addressed the occupational contribution to the burden of airway disease [107]. This statement reviewed the evidence implicating occupational factors in the pathogenesis of the obstructive airway diseases asthma and COPD and tried to quantifythe contribution of the work-related risks to the burden of thesediseases in the general population.

The quantification was performed using an assessment of the population attributable risk (PAR), which is defined as the fraction of cases in a population that arise because ofcertain exposures. By analyzing the data presented on COPD, it can be seen that the disease definition is often based on chronic cough (3 + months of the year) and therefore, some of these studies have indirectly addressed chronic cough. The data of these studies on chronic cough is given in table 1. As the definition of COPD and exposure variedamong the studies analyzed, and there were less studies providing data for an estimate of the PAR of COPD due to occupation than for asthma, there was a greateruncertainty about the estimate but nonetheless, a valueof 15% was reported to be a reasonable estimate of the occupational contributionto the population burden of COPD [107].

**Table 1:** Chronic cough: population-attributable risk due to occupation. Table modified from 107. Abbreviations: ECRHS = European Community Respiratory Health Survey; PAARC = Pollution Atmosphérique et Affections Respiratoires Chroniques/Air Pollution and Respiratory Diseases; PAR% = magnitude of the population attributable risk.

						PAR%			
						
							Calculated by ATS		
								
Disease Definition	Type of Exposure	Study	Sex	Age Range	Subject # Cases #	Reported	1	2	Ref.
**Chronic cough (3+ months of the year)**	Dusts	Population study of six citiesin the U.S.A.	M/F	25–74	8,515/1,015	24	9	8	[102]
	Fumes/gases		M/F		8,515/1,066	23	11	10	
**Chronic cough (cough 3+ months of the year)**	Dusts, gases/fumes	PAARC-Population study of sevenFrench cities	M	29–59	8,692/1,036		11	11	[104]
	Dusts, gases/fumes		F		7,772/407		8	8	
**Chronic bronchitis (as chronic cough and chronic phlegm)**	Dusts	Population study of Cracow followed for 13 years	F	19–70	1,280/175		9	8	[103]
**Chronic cough**	Dusts, fumes, gases	Population study of Po Delta area in North Italy	M	18–64	1,027/159		15	18	[106]
**Chronic cough (cough 3+ months of the year)**	Gases or dusts	Population study of Hordaland county in Norway	M/F	15–70	4,469/409	11	16	15	[117]
**Chronic cough (cough 3+ months of the year)**	Fumes/gases	Population study of three Chinese areas	M/F	40–69	3,606/876		4	4	[118]
	Dusts		M/F		3,606/632		9	9	
**Chronic cough (cough 3+ months of the year)**	Low biologic dusts	ECRHS-Population study of five Spanish areas	M/F	20–44	1,735/248		6	8	[105]

## Conclusion

There is a large amount of epidemiological data pointing to an association between occupational exposure and the prevalence of chronic cough.

However, as chronic cough is often based on a variety of non-occupational but factors such as exposure to tobacco smoke, a distinct separation into either occupational or personally -associated can be difficult. Future experimental and epidemiological studies can lead to a better understanding of the occupational hazards which may cause chronic cough and establish a stronger link between the entity of chronic cough and specific occupations. Therefore, next to enlarging the epidemiological knowledge on the occupational contribution to the disease [108], experimental studies encompassing modern techniques from molecular biology [109,110], physiology [111-113] and morphology [114-116] should be used to identify a cellular basis of work-related chronic cough.

## Declaration of competing interests

The author(s) declare that they have no competing interests.

## Authors' contributions

DAG, DN, AW and AF have all been involved in drafting the article or revising it critically for important intellectual content and have given final approval of the version to be published
